# PTEN in somatostatin neurons regulates fear and anxiety and is required for inhibitory synaptic connectivity within central amygdala

**DOI:** 10.3389/fncel.2025.1597131

**Published:** 2025-06-26

**Authors:** Timothy W. Holford, Kaitlyn N. Letourneau, Carolyn Von-Walter, Daniela Moncaleano, Cody L. Loomis, M. McLean Bolton

**Affiliations:** Disorders of Neural Circuit Function, Max Planck Florida Institute for Neuroscience, Jupiter, FL, United States

**Keywords:** autism, amygdala, PTEN, inhibitory neurons, circuit mapping, synaptic transmission, two-photon microscopy

## Abstract

**Introduction:**

The phosphatase and tensin homolog deleted on chromosome 10 (PTEN) is a negative regulator of the mTOR pathway and is strongly associated with autism spectrum disorder (ASD), with up to 25% of ASD patients with macrocephaly harboring PTEN mutations. Mice with germline PTEN haploinsufficiency show behavioral characteristics resembling ASD, as do various mouse models with conditional knockouts of PTEN. Human tissue studies and those from multiple genetic mouse models suggest that dysfunction of GABAergic interneurons may play a role in the development of ASD, but the precise mechanisms remain elusive. PTEN provides a target for investigation because it regulates the development of inhibitory neurons arising from the medial ganglionic eminence, promoting the survival and maturation of parvalbumin (PV+) neurons at the expense of somatostatin (SOM+) neurons.

**Methods:**

Here, we investigate how PTEN regulates SOM+ neurons at the cellular and circuit level in the central lateral amygdala (CeL), an area that governs the key ASD behavioral symptoms of social anxiety and altered emotional motivation for social engagement using behavioral analysis, electrophysiology, and two-photon local circuit mapping.

**Results:**

We found that knocking out PTEN in SOM+ neurons results in elevated levels of fear and anxiety and decreases CeL local circuit connectivity. Specifically, this manipulation decreased the strength of connections between individual neurons and altered the distribution of local connections in a cell-type specific manner. In contrast to the deficit in local inhibitory connections within CeL, the excitatory drive from the major CeL input, the basolateral amygdala (BLA) was enhanced.

**Discussion:**

This combined imbalance of enhanced excitation and diminished local inhibition likely underlies the heightened fear learning and anxiety we observed in the PTEN-SOM-KO mice.

## 1 Introduction

Autism spectrum disorder (ASD) is a developmental disorder with a prevalence of approximately 1 out of 36 children in the United States (Maenner et al., 2023). The main characteristics are difficulties with social interaction, language or communication deficits, and restricted interests or repetitive behaviors. However, in addition to these, many other co-morbidities may exist, including seizures, anxiety, sensitivity to sensory stimuli, ADHD, cognitive or developmental delays, macroencephaly, and difficulty regulating emotions (Khachadourian et al., 2023).

DNA sequencing studies have identified over 100 ASD risk genes (De Rubeis et al., 2014; Satterstrom et al., 2020; Buxbaum, 2020), and phosphatase and tensin homolog deleted on chromosome 10 (PTEN) is a prominent ASD candidate with PTEN mutations found in up to 25% of ASD patients with macrocephaly (McBride et al., 2010; Varga et al., 2009; Butler et al., 2005; Hobert et al., 2014; Klein et al., 2013; Buxbaum et al., 2007; Frazier et al., 2015).

Phosphatase and tensin homolog deleted on chromosome 10 is expressed throughout the brain and is a potent regulator of cell cycle, growth, and proliferation through its activity as a phosphatase upstream of the AKT/mTOR pathway (Glaviano et al., 2023). The homozygous germline knockout of PTEN is embryonically lethal, while heterozygous mice show behaviors resembling ASD, including deficits in social interaction, anxiety and repetitive behaviors, as well as macroencephaly, hypertrophy of individual neurons, and increased dendritic spine counts (Di Cristofano et al., 1998; Clipperton-Allen and Page, 2014; Clipperton-Allen and Page, 2015; Tilot et al., 2014; Huang et al., 2016). Conditional PTEN knockout (KO) mouse lines have also been generated for various cell types including excitatory and inhibitory neurons (Skelton et al., 2020).

Studies of homozygous PTEN KO in excitatory neurons of the forebrain report macrocephaly, neuronal hypertrophy and, in cases of embryonic deletion, increased dendritic spines and epilepsy [GFAP-Cre (Backman et al., 2001; Fraser et al., 2008); EMX1-Cre (Huang et al., 2016; Chen et al., 2015); NSE-Cre (Kwon et al., 2006; Ogawa et al., 2007; Zhou et al., 2009); CamKIIa-Cre (Sperow et al., 2012; Garcia-Junco-Clemente et al., 2013)]. Neuronal excitability is also affected, but the effect depends on cell type and developmental stage at KO (Garcia-Junco-Clemente et al., 2013; Santos et al., 2017; Williams et al., 2015). In addition to gross morphological and structural changes, PTEN and the target of its phosphatase activity, PIP3, are implicated in long-term depression (LTD) and long-term potentiation (LTP) (Arendt et al., 2010, 2014; Jurado et al., 2010). Consequently, studies of forebrain excitatory neurons with PTEN KOs showed deficiencies in LTP and LTD, and KO of PTEN in dentate granule cells showed increased glutamate release, synapse number and size of postsynaptic responses (Sperow et al., 2012; Luikart et al., 2011; Williams et al., 2015; Tariq et al., 2022).

However, altered inhibition has been hypothesized to play a prominent role in ASD and human patients show altered GABA concentrations and receptor levels in brain regions involved in social processing, as well as altered functional connectivity consistent with an altered excitatory to inhibitory balance (Parrella et al., 2024). Several mouse models of ASD have enhanced or reduced inhibitory function suggesting disruptions to inhibitory networks and excitation/inhibition imbalances have been associated with social deficits in mice (Lee et al., 2017; Yizhar et al., 2011). Importantly, the effect of PTEN deletion on inhibitory neurons is quite different than on excitatory neurons and depends on the type of interneuron. PTEN KO in medial ganglionic eminence (MGE) derived interneurons in NKx2.1 Cre mice decreased the total number of MGE inhibitory neurons but increased the relative number of parvalbumin (PV) to somatostatin (SOM) expressing neurons resulting in a net increase in inhibitory transmission onto cortical pyramidal neurons (Vogt et al., 2015). In addition, conditional PV PTEN (+/−) neurons showed reduced connection probability from PV neurons onto layer 2/3 pyramidal neurons in visual cortex, but the pre and postsynaptic properties of individual connections were not altered (Baohan et al., 2016). Knocking out PTEN in PV or SOM neurons resulted in social deficits, repetitive behaviors and motor impairment, but had strikingly different amygdala-dependent phenotypes, as the SOM KO increased anxiety whereas the PV KO was anxiolytic (Shin et al., 2021).

SOM+ neurons are critical components of cortical and subcortical regions related to ASD behaviors where PV+ activity has been explored (Yizhar et al., 2011; Baohan et al., 2016), but SOM+ specific effects of PTEN loss on ASD-related circuit function have not been previously elucidated. The amygdala is a promising target because it governs emotional behavior known to be disrupted in ASD, including social anxiety and social engagement, and in ASD patients there is evidence of an altered growth trajectory of amygdala nuclei and an altered fMRI response to fearful stimuli (Nordahl et al., 2012; Schumann et al., 2004; Schumann and Amaral, 2006; Kliemann et al., 2010). Similarly, the basolateral amygdala (BLA) has a prominent role in anxiety and is hyperactive in response to social stimuli in PTEN haploinsufficient mice (Huang et al., 2016). Given the hyperactive amygdala and heightened anxiety in the SOM-PTEN-KO, we chose to investigate the functional connectivity in the amygdala of SOM-PTEN-KO mice. We focused on the central lateral amygdala (CeL) because 40% of the neurons in this inhibitory nucleus express somatostatin and because it acts as an inhibitory gate on the downstream expression of fear responses.

We found that the loss of PTEN from SOM+ neurons resulted in increased levels of fear and anxiety as well as drastic reductions in lateral inhibition in the central amygdala. Using two-photon local circuit mapping, we found that knocking out PTEN specifically in SOM+ neurons decreases CeL local circuit connectivity. SOM-PTEN-KO both decreased the strength of connections between individual neurons and altered the distribution of local connections in a cell type specific manner. In contrast to the deficit in local inhibitory connections within CeL, the excitatory drive from the major CeL input, the BLA, was enhanced. This combined imbalance of enhanced excitation and diminished inhibition likely underlies the heightened fear learning and anxiety we observed in the SOM-PTEN-KO mice.

## 2 Results

**FIGURE 1:**
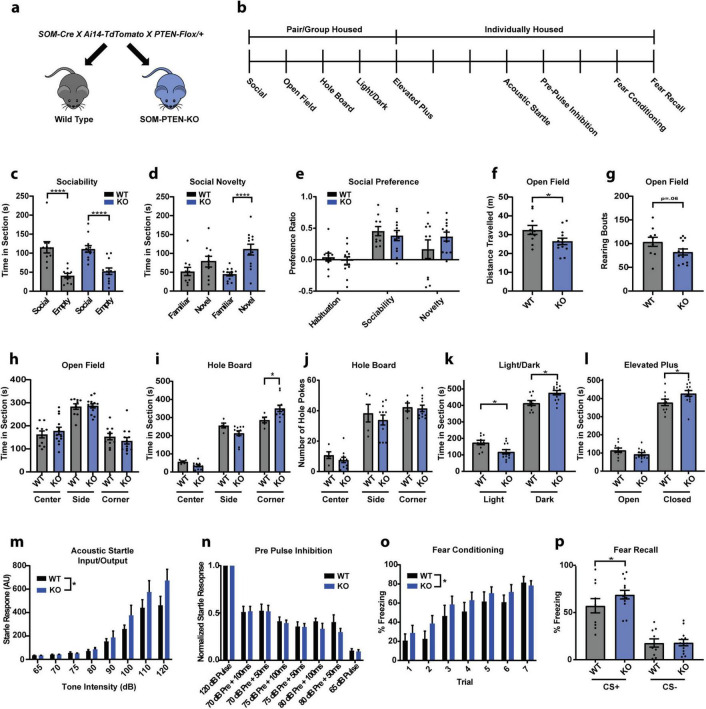
Conditional knockout of PTEN from SOM neurons leads to elevated fear and anxiety phenotype. **(a)** Breeding strategy. **(b)** Experimental timeline for behavior (vertical dashes indicate days). **(c)** Both sets of mice interacted significantly more with social targets than with non-social targets (the empty cup) in the sociability stage (WT: *n* = 10, 115.6 s vs. 41.01 s, **** indicates Tukey’s test: *p* < 0.0001, KO: *n* = 12, 111.2 s vs. 54.65 s, **** indicates Tukey’s test: *p* < 0.0001). **(d)** SOM-PTEN-KO mice showed a higher preference for novel mice, compared to wild type mice, in the social novelty test (WT: *n* = 10, 78.14 s vs. 51.90 s, Tukey’s test: *p* = 0.2526, KO: *n* = 12, 110.0 s vs. 45.20 s, **** indicates Tukey’s test: *p* < 0.0001). **(e)** No significant difference was observed between wild type and knock out mice when comparing their social preference ratios. **(f)** SOM-PTEN-KO mice have reduced locomotion in the open field (*n* = 10 WT and 13 KO, 32.53 m vs. 26.43 m, * indicates *t*-test: *p* = 0.0446). **(g)** SOM-PTEN-KO mice showed a trend toward less exploratory rearing in the open field (*n* = 10 WT and 13 KO, 103.8 vs. 82.38, *p* = 0.0684). **(h)** Wild type and knock out mice show no difference in time exploring the center, edge, or corner zones of the open field. **(i)** SOM-PTEN-KO mice spent more time in the corners during the hole board test [two-way ANOVA for interaction: *F*(2,42) = 7.058, *p* = 0.0023, 286.8 s WT vs. 351.2 s KO, * indicates Sidak’s *post-hoc* test: *p* = 0.013]. **(j)** No differences observed in number or distribution of holes poked. **(k)** SOM-PTEN-KO mice spent less time in the light (174.9 s WT vs. 351.2 s KO, * indicates Sidak’s test: *p* = 0.0133) and more time in the dark (414.3 s WT vs. 477.0 s KO, * indicates Sidak’s test: *p* = 0.0053) compared to WT mice in the light/dark chamber. **(l)** In the Elevated-Plus Maze, knock out mice spent less time in the open arm and more time in the closed arm compared to wild type mice (open arm: 115.5 s WT vs. 93.8 s KO, closed arm: 378.6 s WT vs. 428.2 s KO, * indicates Sidak’s test: *p* = 0.0295). **(m)** Input/output curves reveal an increased startle response in SOM-PTEN-KO mice [*n* = 10 WT and 13 KO mice, two-way ANOVA *F*(1,168) = 5.243, *p* = 0.0211]. **(n)** Normalized PPI shows no difference between wild type and knock out mice in sensory integration [*n* = 10 WT and 13 KO mice, two-way ANOVA – PPI effect: *F*(5,120) = 4.436, *p* = 0.0010, genotype effect: *F*(1,120) = 1.289, *p* = 0.2585]. **(o)** Both genotypes readily acquire fear memory, but SOM-PTEN-KO mice show elevated levels of freezing during conditioning compared to their wild type littermates [*n* = 10 WT and 13 KO mice, two-way ANOVA – conditioning effect: *F*(6,147) = 12.01, *p* = 0.0001, * indicates genotype effect: *F*(1,147) = 4.480, *p* = 0.0360]. **(p)** Both knock out and wild type mice can discriminate between CS+ and CS−, but SOM-PTEN-KO mice freeze more to the CS+ (*n* = 10 WT and 13 KO mice, 57.17% vs. 68.91%, * indicates nested *t*-test: *p* = 0.0364).

Removing PTEN selectively in postnatal somatostatin neurons has been shown to be sufficient for the expression of ASD behaviors such as social deficits, repetitive behaviors, and anxiety (Shin et al., 2021). To determine the role of PTEN in somatostatin-expressing neurons and confirm the effect of removing PTEN from these neurons on ASD-related behaviors, we created a triple cross of SOM-Cre; Ai14-TdTomato; PTEN-Flox mice to knock out PTEN specifically from SOM+ neurons (Figure 1a). We then tested wild type and homozygous somatostatin PTEN knockout (SOM-PTEN-KO) mice on a variety of ASD associated behaviors (Figure 1b).

### 2.1 Conditional SOM-PTEN-KO mice have heightened fear and anxiety but normal social behavior and no tendency for enhanced repetitive behavior

Overall, SOM-PTEN-KO mice showed behavioral differences in tasks related to exploration, anxiety and fear, but not in the full repertoire of ASD-related behaviors. In contrast to many ASD-models that show social deficits, social interaction and social novelty remained intact in SOM-PTEN-KO mice (Figures 1c–e). SOM-PTEN-KO mice displayed less exploratory behavior in an open field task, but their time in center and edge zones was not different than wild type mice (Figures 1f–h). In contrast, SOM-PTEN-KO mice showed indications of anxiety-like responses in the hole board test of investigative behavior, as SOM-PTEN-KO mice spent more time in the corners of the arena when the hole board was present, suggesting a tendency for anxiety-like behavior in more stimulating environments, though they did not display restricted-repetitive motion as measured by repetitive hole-poking (Figures 1i, j). SOM-PTEN-KO mice also showed increased levels of anxiety in both the light/dark test and the elevated plus maze (EPM) (Figures 1k, l, respectively). Altered sensory perception is common in ASD patients (Balasco et al., 2020), and similarly, SOM-PTEN-KO mice showed an enhanced sensitivity to sensory stimuli, measured here by startle response to presentations of white noise of various intensities (Figure 1m). However, sensory integration measured by pre-pulse inhibition (PPI) was unaffected (Figure 1n). Fear behavior was also elevated in SOM-PTEN-KO mice compared to wild type mice. SOM-PTEN-KO mice acquired fear memory faster as they froze more to tones paired with a mild foot-shock during differential fear conditioning (Figure 1o), and they had higher levels of freezing during fear recall, with the increased freezing specific to the CS+ tone and not generalized to CS− (Figure 1p).

### 2.2 Increased AMPA/NMDA synaptic current ratio at basolateral amygdala to central lateral amygdala somatostatin neuron synapse in SOM-PTEN-KO mice

**FIGURE 2:**
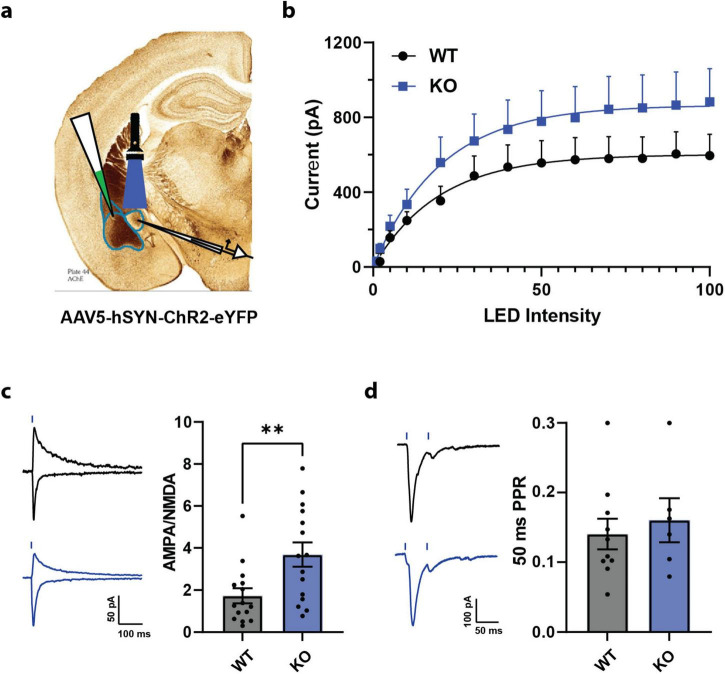
SOM+ PTEN knockout disrupts BLA to CeL signaling. **(a)** Experimental strategy for recording BLA afferents onto SOM+ neurons in the CeL. **(b)** Opsin-induced excitatory currents onto CeL SOM+ cells trend larger in knock out mice, but are not statistically significant [*n* = 18 WT and 17 KO cells, mixed effects ANOVA – genotype: *F*(1,33) = 1.922, *p* = 0.1749, max current mean: 570.2 pA WT vs. 849.5 pA KO, *t*-test: *p* = 0.1743]. **(c)** (Left) Representative traces from WT (black) and KO (blue) cells, scale bar is 50 pA = 100 ms, blue lines indicate optogenetic stimulation, (right) AMPA/NMDA ratio is higher in knock out mice (*n* = 15 WT cells, mean = 1.73 and 15 KO cells, mean = 3.68, ** indicates *t*-test: *p* = 0.0077). **(d)** (Left) Representative traces from WT (black) and KO (blue) cells, scale bar is 100 pA = 50 ms, blue lines indicate optogenetic stimulation, (right) no difference between WT and KO mice was observed when comparing PPR (*n* = 10 WT cells, mean = 0.141, and 6 KO cells, mean = 0.160, *t*-test: *p* = 0.6051).

The behavioral consequences of the conditional knockout of PTEN only in SOM+ neurons were heavily represented in tasks relating to the regulation of fear and anxiety. As such, we focused our morphological and physiological investigation of the SOM-PTEN-KO mice on the amygdala, which has long been associated with emotional regulation and the expression of fear and anxiety (Janak and Tye, 2015). To assess the physiological effects of the conditional knockout of PTEN, and to find potential cellular correlates of the aberrant behavior seen in SOM-PTEN-KO mice, we turned to acute-slice patch clamp electrophysiology. Emotionally relevant sensory information enters the amygdala through the lateral amygdala (LA) which in turn projects to the basal amygdala (BA). The BA also receives association information directly from higher order structures. SOM neurons in CeL receive excitatory input from LA and BA (collectively termed BLA) and this connection from BLA to CeL neurons is essential for the regulation of fear and anxiety-related behaviors (Li, 2019; Fadok et al., 2017). To investigate whether these inputs onto CeL SOM+ neurons were disrupted by the loss of PTEN, we injected the BLA with an AAV expressing channelrhodopsin (ChR) and patched SOM+ neurons in the CeL (Figure 2a). First, we established an input/output curve by recording post synaptic responses to different levels of LED intensity. Overall, SOM-PTEN-KO SOM+ cells showed an increased, though not statistically significant, peak excitatory current compared to wild type cells across the input/output curve (Figure 2b). We then measured the AMPA/NMDA ratio and paired-pulse ratio to see if SOM-PTEN-KO SOM+ cells had post synaptic or presynaptic effects, respectively. We found that the AMPA/NMDA ratio was significantly increased in SOM-PTEN-KO SOM+ cells compared to wild type (Figure 2c). On the other hand, the paired-pulse ratio was not affected by loss of PTEN (Figure 2d). The combination of the trend toward increased amplitude and increased AMPA/NMDA ratio suggests enhanced AMPA mediated excitatory drive to CeL SOM+ neurons.

### 2.3 Two-photon local circuit mapping in the central amygdala using GCaMP6m and a soma-restricted ChRmine

**FIGURE 3:**
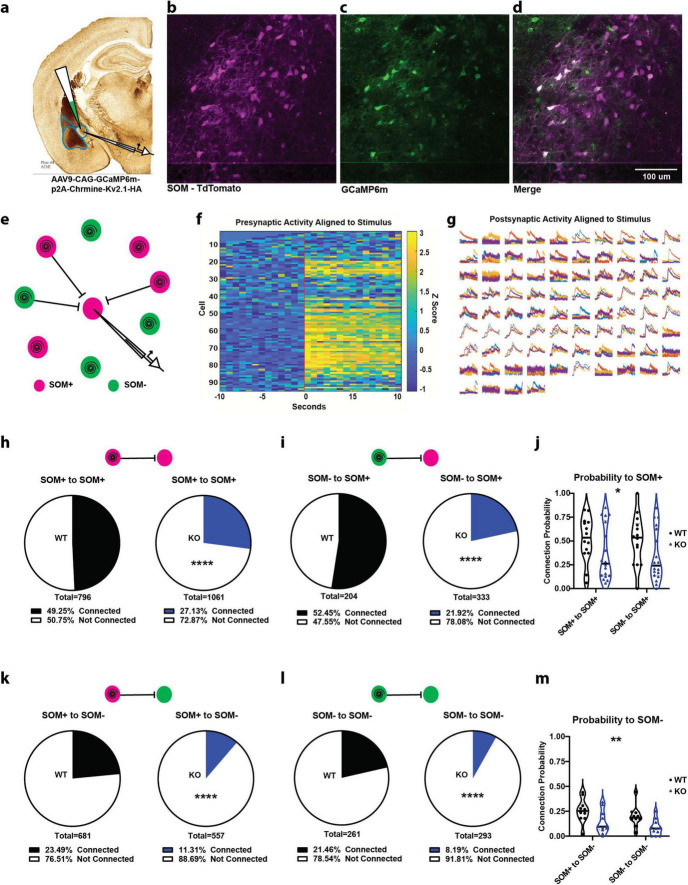
SOM+ PTEN knockout drastically reduces connection probability within the CeL. **(a)** Experimental strategy for recording connections within the CeL. **(b–d)** Example images of **(b)** tdTomato expression (SOM+ neurons), **(c)** GCaMP/ChRmine expression (optogenetic target cells), and **(d)** merged images. **(e)** Schematic of circuit mapping strategy, patching a cell in the center of the FOV to measure optically induced postsynaptic currents and spiral scanning to activate surrounding neurons (magenta circles indicate SOM+ cells expressing tdTomato and GCaMP/ChRmine, green cells indicate SOM+ cells expressing only GCaMP/ChRmine). **(f)** Example of *z*-scored, time-locked GCaMP fluorescence changes in optically targeted cells, vertical red bar indicates onset of activation. **(g)** Example of time-locked postsynaptic current recorded from the patched cell, each subplot represents activation of one of the 94 presynaptic targets in **(f)**, and colors represent one of four repetitions. **(h)** SOM+ to SOM+ (*n* = 392 connections out of 796 activated presynaptic cells onto 14 patched WT cells, proportion connected = 49.25 − 3.46% vs. 288 connections out of 1,061 activated presynaptic cells onto 18 patched KO cells, proportion connected = 27.13 +/− 2.67%, **** indicates difference of proportions test, *p* < 0.0001). **(i)** SOM− to SOM+ (*n* = 107 connections out of 204 activated presynaptic cells onto 14 patched WT cells, proportion connected +/− 52.45 − 6.79% vs. 73 connections out of 333 activated presynaptic cells onto 18 patched KO cells, proportion connected = 21.92 +/− 4.43%, **** indicates difference of proportions test, *p* < 0.0001). **(j)** SOM-PTEN-KO mice also show reduced average connection probability by cell [*n* = 14 postsynaptic WT SOM+ cells, mean = 0.5132, and 18 postsynaptic KO SOM+ cells, mean = 0.3557, * indicates two-way ANOVA genotype Effect: *F*(1, 60) = 5.528, *p* = 0.022]. **(k)** SOM+ to SOM+ (*n* = 160 connections out of 681 activated presynaptic cells onto 10 patched WT cells, proportion connected = 23.49 +/− 3.18% vs. 63 connections out of 557 activated presynaptic cells onto 11 patched KO cells, proportion connected = 11.31 +/− 2.63%, **** indicates difference of proportions test, *p* < 0.0001). **(l)** SOM− to SOM+ (*n* = 56 connections out of 261 activated presynaptic cells onto 10 patched WT cells, Proportion connected = 21.46 +/− 4.80% vs. 24 connections out of 293 activated presynaptic cells onto 11 patched KO cells, proportion connected = 8.19 +/− 3.17%, **** indicates difference of proportions test, *p* < 0.0001). **(m)** SOM-PTEN-KO mice also show reduced average connection probability by cell [*n* = 10 postsynaptic WT SOM+ cells, mean = 0.2260, and 11 postsynaptic KO SOM+ cells, mean = 0.1100, ** indicates two-way ANOVA genotype effect: *F*(1,38) = 8.809, *p* = 0.0052].

SOM+ neurons in CeL, when driven by emotional stimuli through BLA, inhibit a second class of inhibitory neurons within CeL, the SOM− (PKCδ+) neurons, and also inhibit neighboring SOM+ neurons. The CeL then projects to the central medial amygdala (CeM) which sends inhibitory projections to the downstream effectors of emotional responses. After investigating BLA inputs onto SOM+ neurons, we wanted a detailed look into the effects of SOM-PTEN-KO on local inhibitory signaling within the CeL. To this end, we conducted dual two-photon and electrophysiological, local circuit mapping experiments to probe single-neuron connectivity. This was enabled in part by the restriction of the high-efficiency, red-shifted opsin, Chrmine, to express only in the soma and proximal dendrites via a targeting sequence from the potassium channel Kv2.1, which dramatically reduces off-target neuronal activation and has been done with other opsins (Baker et al., 2016; Marshel et al., 2019). Chrmine is ideally suited for the two-photon spiral scan method of single cell activation due to its large conductance and slow deactivation kinetics allowing integration of the activity of the opsin over the entire area of the soma illuminated by the spiral to fire the neuron (Marshel et al., 2019). This opsin was co-expressed with GCaMP6m, by delivery on the same AAV construct, to the CeL (Figure 3a).

A typical local circuit mapping experiment was conducted by patching a single neuron in the CeL, identifying potential presynaptic partners expressing GCaMP6m under 2P imaging (Figures 3b–d), and simultaneously recording electrophysiological currents in voltage clamp and 2P GCaMP6m fluorescence as each presynaptic target was activated in turn by the stimulation laser (Figures 3e–g). Connections were determined by coincident increase in GCaMP6m fluorescence (for presynaptic cells) and inhibitory postsynaptic current (for patched postsynaptic cells), and targeted cells that showed a presynaptic calcium transient with no corresponding postsynaptic current were deemed activated but not connected. We could then move the FOV to a different Z plane (30–50 μm deeper) and repeat the targeting, imaging, and recording for a separate set of presynaptic cells onto the same patched postsynaptic neuron. This method allowed us to evaluate the presence and strength of single-cell connectivity from hundreds of presynaptic cells onto one patched neuron, an efficiency that is orders of magnitude higher than paired-patch techniques.

### 2.4 SOM+ PTEN KO leads to decreased CeL local circuit connectivity

Overall, SOM-PTEN-KO drastically reduced the level of local inhibitory connectivity in the central amygdala by around 50% (Figures 3h–m).

First, we looked at connections onto SOM+ cells in wild type and SOM-PTEN-KO mice (Figures 3h–j). In wild type mice, SOM+ neurons showed a connection probability of 49.25% to other SOM+ neurons. Inhibitory post synaptic currents were detected from 392 out of the 796 cells that were activated by the stimulation laser and had presynaptic firing confirmed by GCaMP6 fluorescence (Figure 3h). SOM+ neurons in SOM-PTEN-KO mice, on the other hand, showed a reduced connection probability of just 27.13% to other SOM+ neurons. Inhibitory post synaptic currents were observed from 288 out of 1,061 spike-confirmed presynaptic targets (Figure 3h). Connection probability was also measured from SOM− neurons onto SOM+ neurons. In wild type mice, SOM− to SOM+ neurons showed a connection probability of 52.45% (107/204) compared to a reduced probability of 21.92% (73/333) in SOM-PTEN-KO mice (Figure 3i). Similarly, the average number of connections per post-synaptic cell is lower in SOM-PTEN-KO neurons compared to wild type neurons across all conditions (Figure 3j).

We also mapped the local circuit connectivity onto postsynaptic SOM− neurons within the CeL and found it was drastically reduced in SOM-PTEN-KO mice (Figures 3k–m). For wild type mice, SOM+ to SOM− neurons showed a connection probability of 23.49% (160/681). In contrast, SOM-PTEN-KO mice had a connection probability of just 11.31% (63/557) for SOM+ to SOM− neurons (Figure 3k). We found a similar result in SOM− to SOM− connectivity. In wild type mice, SOM− to SOM− neurons showed a connection proportion of 21.46% (56/261), while in SOM-PTEN-KO mice, there was a probability of connection of 8.19% (24/293) (Figure 3l). In summary, the average number of connections per cell is lower in SOM-PTEN-KO neurons compared to wild type neurons across all conditions (Figure 3m).

### 2.5 SOM+ PTEN KO decreases strength and alters distribution of local connections in a cell type specific manner

**FIGURE 4:**
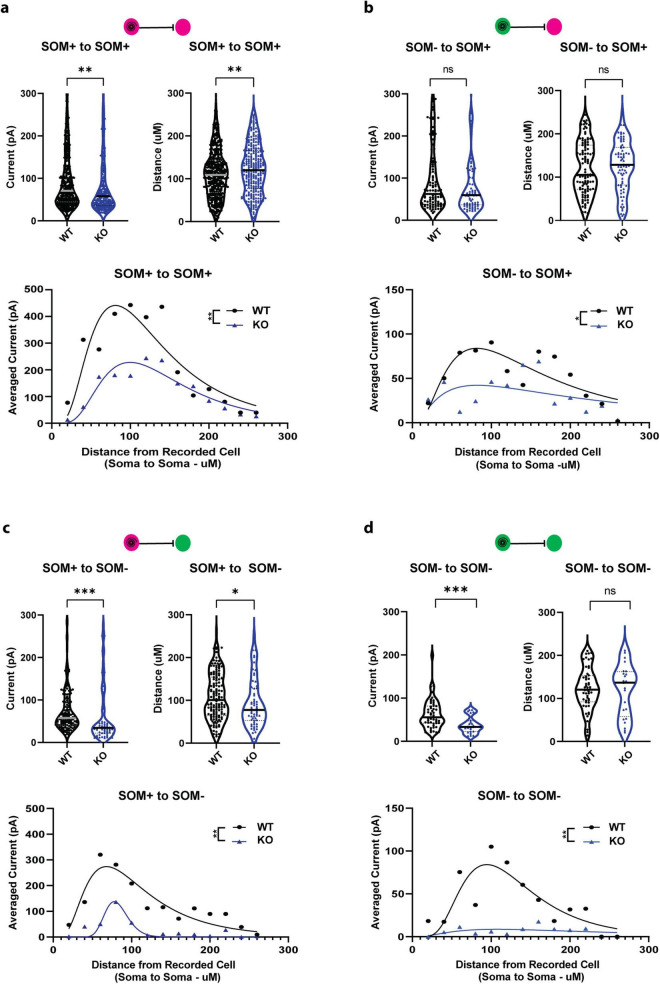
Strength and distribution of CeL inhibitory currents are disrupted in SOM-PTEN-KO mice. **(a)** SOM+ to SOM+ (*n* = 392 connections onto 14 patched WT cells and 288 connections onto 18 patched KO cells): (top left) individual postsynaptic currents are reduced in knock out mice (median: 70.6 pA vs. 57.9 pA, ** indicates Mann–Whitney test: *p* = 0.0037), (top right) individual postsynaptic currents originate further away in knock out mice (median: 108.9 μm vs. 120.1 μm, ** indicates Mann–Whitney test: *p* = 0.0052), (bottom) the distribution of averaged current density, binned by distance from the presynaptic cell, is reduced and shifted further away in knock out mice [** indicates two-way ANOVA genotype effect: *F*(1,12) = 13.42, *p* = 0.0032, curves represent lognormal fit for visualization]. **(b)** SOM+ to SOM+ (*n* = 107 connections onto 14 patched WT cells and 73 connections onto 18 patched KO cells): (top left) no difference in strength of individual currents in wild type vs. knock out mice (median: 62.3 pA vs. 59.7 pA, Mann–Whitney test: *p* = 0.6172), (top right) no difference in distance for individual currents in wild type vs. knock out mice (median: 105.2 μm vs. 128.3 μm, Mann–Whitney test: *p* = 0.4858), (bottom) average current density distribution is reduced in knock out mice [two-way ANOVA genotype effect: *F*(1,12) = 7.651, *p* = 0.0171, curves represent lognormal fit for visualization]. **(c)** SOM+ to SOM− (*n* = 160 connections onto 10 patched WT cells and 63 connections onto 11 patched KO cells): (top left) individual currents are reduced in knock out mice (median: 52.6 pA vs. 34.3 pA, *** indicates Mann–Whitney test: *p* = 0.0003), (top right) connections to SOM− cells are closer in knock out mice (median: 94.1 μm vs. 77.7 μm, * indicates Mann–Whitney test: *p* = 0.0436), (bottom) average current density distribution is reduced in knock out mice [** indicates two-way ANOVA genotype effect: *F*(1,12) = 17.94, *p* = 0.0012, curves represent lognormal fit for visualization]. **(d)** SOM− to SOM+ (*n* = 56 connections onto 10 patched WT cells and 24 connections onto 11 patched KO cells): (top left) individual currents are reduced in knock out mice (median: 53.7 pA vs. 33.2 pA, *** indicates Mann–Whitney test: *p* = 0.0008), (top right) no difference in distance for individual currents in wild type vs. knock out mice (median: 120.3 μm vs. 136.7 μm, Mann–Whitney test: *p* = 0.9460), (bottom) average current density distribution is reduced in knock out mice [** indicates two-way ANOVA genotype effect: *F*(1,12) = 13.67, *p* = 0.0031, curves represent lognormal fit for visualization].

In order to investigate the effects of documented cell overgrowth as a result of PTEN deletion, which could alter local circuit geometry and computation, we evaluated the strength and distribution of individual post-synaptic currents recorded in SOM-PTEN-KO mice compared to wild type mice. Individual currents from SOM+ to SOM+ neurons were smaller and originated further away, as measured by soma-to-soma distance, in SOM-PTEN-KO mice compared to wild type (Figure 4a, top). To assess the cumulative effects of reduced currents, as well as the spatial distribution of their presynaptic origins, we binned IPSCs based on their soma-to-soma distance in 20 μm concentric circles, similar to Sholl analysis (Sholl, 1953), then divided by the total number of post-synaptic neurons for each configuration. When pooled together, the average distribution of total post-synaptic current reveals cumulative differences in the current-distance relationship onto SOM+ neurons, which could disrupt local circuit computation (Figure 4a, bottom). Individual currents recorded from SOM− to SOM+ neurons were not significantly smaller or further away (Figure 4b, top), however, the cumulative effects of fewer connections are still observed (Figure 4b, bottom).

Individual currents onto SOM− neurons were also altered in the SOM-PTEN-KO mice. The strength in post-synaptic currents recorded from SOM+ to SOM− neurons was reduced, but in this case the average distance from which the neuron received inputs was also reduced (Figure 4c, top) with a greatly reduced current distribution (Figure 4c, bottom). Recordings from SOM− to SOM− showed weaker synaptic currents (Figure 4d, top) and a drastic reduction in current distribution even though the expression of PTEN was not altered in either the pre-or postsynaptic neuron (Figure 4d, bottom).

## 3 Discussion

**FIGURE 5:**
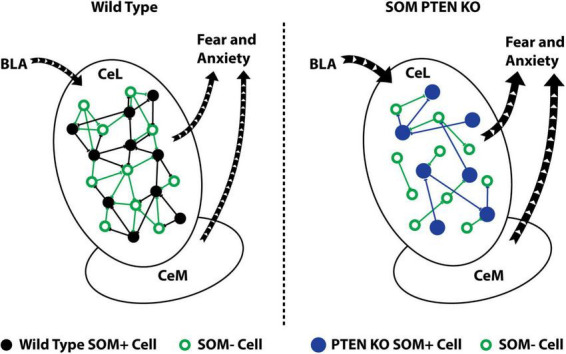
SOM+ PTEN KO alters local circuit connectivity and leads to increased fear and anxiety. Schematic summary of the effects of SOM-PTEN-KO: in wild type mice, the CeL receives input from the BLA that activates an intact local inhibitory network and leads to appropriate levels of fear and anxiety **(left)**, while in SOM-PTEN-KO mice, input from the BLA activates the CeL, which has reduced levels of lateral inhibition, and leads to excess fear and anxiety **(right)**.

Here, we investigated the role of PTEN in somatostatin neurons in amygdala circuits, confirming that KO of PTEN in SOM+ neurons is sufficient to increase anxiety and expanding the behavioral phenotype by demonstrating elevated fear and a heightened acoustic startle response. We further revealed mechanistically relevant microcircuit alterations underlying the behavioral phenotype by using electrophysiology and cellular-resolution two-photon circuit mapping, finding increased excitatory drive to SOM+ neurons and decreased CeL local circuit connectivity (Figure 5). These findings provide further evidence of amygdala dysfunction in models of ASD, and highlight the importance of functional circuit mapping to correlate behavioral deficits with genetic mutations.

This study is an in-depth characterization of circuitry dysfunction within the CeL and correlation with behavioral phenotype in a conditional PTEN KO model of ASD. While many genes have been shown to affect the development and progression of ASD, we chose to investigate circuit changes in PTEN KO models due to the high confidence of PTEN as an ASD risk gene, prevalence of PTEN mutations in ASD patients with macrocephaly, and established ASD behavioral phenotype in the germline PTEN haploinsufficient mouse model. In addition, PTEN regulates the function of several other ASD risk genes within the PI3K/AKT/mTOR pathway such as TSC1/2 (Asano et al., 2001; de Vries et al., 2005) and NF1 (Mbarek et al., 1999). While conditional KO models do not represent the genetic state of ASD patients, they provide insights into the contribution of the risk gene in specific cell types or developmental periods to changes in cellular function, network connectivity and behavioral changes. We focused on SOM-PTEN-KO because the number of SOM+ neurons is reduced in the Nkx2.1 Cre MGE conditional PTEN KO model, showing a role for PTEN in the early development of inhibitory neurons from neuronal progenitors (Vogt et al., 2015) and because SOM-PTEN-KO is sufficient for the expression of an ASD behavioral phenotype with a strong anxiety component (Shin et al., 2021). Additionally, a postnatal knock out of PTEN from excitatory neurons revealed disruptions to some of PTEN’s effects on neuronal plasticity and signaling without affecting gross morphology or broad cell overgrowth, indicating a morphological and migratory role for PTEN during embryonic development, but continued synaptic importance throughout the lifetime (Sperow et al., 2012). By using the SOM-Cre line to knock out PTEN from SOM+ cells during development starting at approximately E12.5 (Taniguchi et al., 2011), we can identify that the formation of local circuitry depends on PTEN activity in SOM+ cells as well as how the persistent lack of PTEN from SOM+ cells continues to impact neuronal signaling and behavior into adulthood.

Shin et al. (2021) showed that mice with SOM-PTEN-KO had increased anxiety, repetitive behavior, decreased locomotion, deficits with motor coordination, normal sociability but decreased social novelty preference. We confirmed the Shin et al. (2021) finding that the conditional SOM-cell PTEN knock out mice showed elevated levels of anxiety-related behaviors. In addition, we showed that SOM-PTEN-KO increased fear learning and sensory sensitivity (Figure 1). Our findings broaden the scope of behavioral disruptions in SOM-PTEN-KO mice as well as the role that SOM+ neurons play in regulating ASD-related behaviors. Dysregulation of fear, anxiety, and sensory sensitivity has also been observed in some PTEN models (Kwon et al., 2006; Vogt et al., 2015; Clipperton-Allen and Page, 2014) as well as in other mouse models of ASD such as SHANK3 (Peça et al., 2011; Jiang and Ehlers, 2013) and CNTNAP2 (Peñagarikano et al., 2011). The social impairments seen in constitutive PTEN (+/−) mice were not observed in our conditional SOM-cell PTEN KO mice, suggesting that the circuits for regulating social interactions are less dependent on SOM+ neuron-specific activity, or that SOM+ dependent activity has been compensated for by other mechanisms. The lack of effect on social novelty preference in our study is in contrast to the decreased social novelty preference observed by Shin et al. It is not clear why our results diverge but likely is due to differences in the test protocol or ambient conditions during testing. While the strain and driver lines for both studies are the same, they used mice a few weeks younger than ours as well as a different apparatus geometry and detection method for social interaction.

The central amygdala is composed of several subnuclei that regulate the behavioral expression of fear and emotions. The CeL receives excitatory inputs conveying information about aversive stimuli from LA, paraventricular nucleus of the thalamus, insular cortex, ventral hippocampus and parabrachial nucleus (Ahrens et al., 2018). Information then cascades down a series of inhibitory synapses within the CeL to disinhibit the CeM output neurons. The CeM in turn projects to areas in the brain stem and hypothalamus regulating autonomic and action selection responses (Fadok et al., 2018). In the classic cued fear conditioning paradigm, excitatory inputs from BLA synapse onto SOM+ neurons in CeL. The SOM+ neurons then inhibit the SOM− (PKCδ+) neurons, leading to disinhibition of CeM neurons projecting to areas inducing the freezing response. In addition, SOM+ neurons in CeL project directly to the PAG to contribute to the freezing response (Massi et al., 2023; Penzo et al., 2014). Plasticity representing the learning of a fear association occurs at multiple synapses along the amygdala information processing stream. Association of the US and CS are represented by plasticity of excitatory synapses onto LA and BA from cortical and thalamic areas. However, studies have shown the importance of plasticity at excitatory synapses onto CeL inhibitory neurons in selecting and strengthening appropriate fear response actions (Penzo and Moscarello, 2023). In the current study, we focused on the CeL for mechanistically relevant microcircuit changes because over 40% of the neurons within this inhibitory nucleus are SOM+ with most of the remaining SOM− neurons expressing PKCδ+. The CeL, with such a large population of SOM+ cells, would likely be disturbed by a SOM-specific knockout and provided a clear target for investigating PTEN-dependent local circuit functional connectivity.

We identified mechanistic circuit-level disruptions that may underlie the behavioral phenotype observed in the conditional PTEN knock out mice using optogenetically activated axon terminals (Figure 2) and a modified version of our two-photon assisted circuit mapping technique (Figure 3). We have demonstrated previously that using a Kv2.1-tagged opsin restricts its expression to the soma and proximal dendrites and allows for robust detection of cell activity when paired with a genetically encoded calcium indicator and two-photon imaging (Baker et al., 2016). Specifically, the two-photon imaging enhances optical sectioning, limiting activation of opsins outside the field of view (FOV), and the soma-restriction of the opsin reduces the activation of off-target neurons’ overlapping dendrites or axons, providing single-cell specificity. This two-photon assisted circuit mapping technique has been adapted and applied to numerous other brain areas to understand local circuit functional connectivity (Anastasiades et al., 2021). We found that SOM+ cells lacking PTEN in the CeL showed enhanced excitatory drive from BLA but disrupted patterns of local inhibitory synaptic connectivity and synaptic strength compared to wild-type SOM+ neurons, which conceivably underlies the increased levels of fear and anxiety observed in our mice.

Fear conditioning has been shown to increase the strength of the BLA to SOM+ connection (Ciocchi et al., 2010; Hartley et al., 2019). We observed an increase in the basal strength of the BLA to CeL SOM+ neurons and an increased AMPA to NMDA ratio in the SOM-PTEN-KO. The AMPA/NMDA ratio is a measure of the postsynaptic receptor composition and changes in this ratio reflect many underlying activity dependent and/or developmental processes such as receptor trafficking and synaptic scaling (de León-López et al., 2025). It is not clear if this is the result of accumulated strength due to increased propensity for LTP at the BLA to CeL SOM+ synapse during fear learning or a constitutive increase in strength of this synapse, though it could be a combination of both. The target of PTEN’s phosphatase activity, PIP3, is important for regulating postsynaptic AMPA receptor clustering, and this would be strengthened with the loss of PTEN, which could support increased AMPA-mediated synaptic currents (Arendt et al., 2010). Similarly, PTEN KO in excitatory forebrain neurons disrupts both LTD and LTP at excitatory synapses onto dentate granule and CA1 pyramidal neurons (Sperow et al., 2012; Takeuchi et al., 2013) and PTEN has been shown to have a bidirectional effect on plasticity in LA neurons, with reduced PTEN activity diminishing the amount of LTD (Sánchez-Puelles et al., 2020; Jurado et al., 2010). Notably, those studies recorded from excitatory neurons, whereas with the present SOM-PTEN-KO, the presynaptic excitatory synaptic properties were not affected, as seen in the lack of presynaptic PPR differences. The direct effect of SOM-PTEN-KO on plasticity at the BLA to SOM+ CeL synapse will need to be addressed in future experiments, and a recently developed PTEN sensor for fluorescent lifetime imaging (FLIM) may enable further investigation into PTEN’s functional role in synaptic compartments (Kagan et al., 2025).

Within the CeL, the SOM-PTEN-KO mice had drastically reduced connectivity. The probability of connection between any two groups of neurons in the SOM-PTEN-KO mice (SOM+ to SOM−, SOM+ to SOM+, SOM− to SOM+, SOM− to SOM−) was about 50% of that observed in wildtype mice. The SOM− to SOM− cell effects likely are the result of compensatory efforts by local networks to account for the reduction of SOM+ activity. With reduced inputs from SOM+ neurons, the lateral inhibition required from SOM− to respond to incoming activity may have also reduced. While the precise mechanisms of the non-cell autonomous effects remain elusive, there is evidence for homeostatic and activity-dependent plasticity in inhibitory networks (Tien and Kerschensteiner, 2018; Flores and Méndez, 2014). For example, chronic blockades of activity onto interneurons in both the hippocampus and cortex have resulted in net decreases of inhibitory signaling afterward (Bartley et al., 2008; Jiao et al., 2006; Hartman et al., 2006), and though these are in circuits with primarily excitatory afferents, GABA signaling has been shown to also activate activity-dependent postsynaptic response elements both in inhibitory networks and developmental circuits (Tu et al., 2007; Zhang et al., 2015; Jagasia et al., 2009), so the mechanism of postsynaptic inhibitory reduction may be the same. Future studies, perhaps involving computational models, may shed more light on these compensatory changes and their effects on network activity.

In addition, the strength of connection from SOM+ to both the SOM+ and SOM− neurons was reduced. In the case of wildtype mice, the connection probabilities are similar to those reported by Hunt et al. (2017) in their paired patch study. In both the current study and Hunt et al., SOM+ are highly connected with other SOM+ neurons (49% current study vs. 56% in Hunt). Like Hunt, we found that the connection probability from SOM− to SOM− neurons is less than between SOM+ pairs (22% current study vs. 21% Hunt). However, we found that SOM− to SOM+ pairs were highly connected whereas Hunt found a lower probability of connection (52% current study vs. 22% Hunt). In our experiments SOM+ had a moderate connection probability onto SOM− neurons but higher than that observed by Hunt (23% current study vs. 12% Hunt). The higher probability of connections in our experiments compared to the Hunt study likely reflect the larger soma-to-soma distance we were able to sample with our circuit mapping method compared to paired patching.

While it is not possible to determine the net behavioral effect of reducing the connectivity at each of these classes of synapses in this complex network of feed forward and feedback inhibitory loops, previous pharmacological and optogenetic experiments provide some context. Pharmacological inhibition of CeL or optogenetic activation of CeM induces freezing (Ciocchi et al., 2010). Likewise, chemogenetic inhibition of PKCδ+ neurons promotes freezing (Haubensak et al., 2010) whereas optogenetic activation of SOM+ induces freezing (Li et al., 2013). The increased fear and anxiety behavioral phenotype we observed is consistent with increased activity of SOM+ neurons due to the combination of increased excitatory drive and reduced lateral inhibition from neighboring SOM+ and/or SOM− (PKCδ+) neurons. Although reduced inhibition from SOM+ to SOM− (PKCδ+) neurons could theoretically disinhibit the SOM− (PKCδ+) neurons, this is not the dominant behavioral outcome as this would tend to oppose freezing. This could be because the excitatory drive to SOM− (PKCδ+) neurons arises from distinct sources and is involved in different behavioral actions. The BLA SOM+ input stream instigates freezing whereas the BLA SOM− (PKCδ+) stream promotes avoidance escape behaviors (Penzo and Moscarello, 2023).

Phosphatase and tensin homolog deleted on chromosome 10 has been shown to negatively regulate the cell cycle transition from growth and proliferation (Groszer et al., 2006), which leads to broad overgrowth in its absence, and tumors in many tissues. However, in the brain, this morphological growth does not always result in functional connectivity. Several groups have observed larger cells and increased dendritic and axonal arborization and size, as well as increased numbers of dendritic spines in various PTEN knockouts, though it is not always paired with investigation of functional synapses. Our results indicate a decrease in *functional* inhibitory synapses between CeL neurons, which could reflect a decrease in structural synapse number, or the number of those synapses that are functional in terms of presynaptic GABA release and postsynaptic GABA receptor function. With respect to excitatory transmission, there is a precedent of PTEN KO increasing spine density but also altering their morphology in pyramidal neurons (Kwon et al., 2006; Luikart et al., 2011). LM or EM studies would be required to determine if the number of structural inhibitory synapses was reduced in CeL neurons. Importantly, ultrastructural studies have shown that even in the presence of more numerous axonal boutons and dendritic spines in PTEN KO cells, the molecular machinery important for functional synapses can be markedly absent (Fraser et al., 2008). Although patch-clamp electrophysiology provides the most sensitive method for detecting functional synaptic currents, the response amplitude distribution resulting from unitary vesicles of GABA release does not completely resolve from the noise envelope of the recordings. This implies that a difference in detection between two conditions could theoretically affect results. In this study, the recorded neurons do have a higher capacitance, likely reflecting their larger size which could affect electrotonic filtering of synaptic currents, but the mean unitary connection amplitude in both cases is well above the noise detection limit so we do not anticipate the reduced connectivity to be a result of potential minor differences in detection thresholds.

It is probable that the observed decrease in functional inhibitory connectivity with CeL results from a combination of reduced synapse number and reduced synaptic function of individual synapses, which further highlights the impact of large-scale functional circuit mapping employed here to identify region-wide, local, unitary neuronal connections with physiological relevance in addition to their morphological structure.

Taken together, our work shows that the deletion of PTEN from SOM-expressing interneurons results in disruptions to local inhibitory signaling in the central amygdala and gives rise to fear and anxiety-related deficits that are common in ASD. These projects, and future studies following the same lines, will help to differentiate the roles of specific cell-types and local circuits in neurological disorders, and may pave the way for future therapeutic interventions that can more accurately target isolated behavioral phenotypes.

## 4 Materials and methods

### 4.1 Animals

All animals were bred, housed and maintained in accordance with protocols approved by the IACUC at MPFI, and all surgical procedures and euthanasia were performed in line with those same protocols. We generated conditional knockouts of PTEN by triple crossing *PTEN-Flox* (JAX Stock No. 006440), *SOM-Cre* (JAX Stock No. 013044), and *Ai14-RosaTdTomato* (JAX Stock No. 007909) mice (The Jackson Laboratory, Bar Harbor, ME, USA). This ensured that PTEN would only be missing from cells expressing somatostatin, and those cells would also express the genetically encoded fluorescent marker, TdTomato. Littermate controls were used in all experiments as the PTEN Flox/+ parents generated +/+, FL/+, and FL/FL pups. Male and female mice, ages 9–12 weeks old, were used in all experiments.

All animals were pair- or group- housed for social, open field, hole board, light/dark, and EPM tasks and single-housed for acoustic startle, PPI and fear conditioning/recall. Mice were handled for 3 days prior to testing and allowed to habituate to the testing room for 1 h prior to each behavioral experiment. We tested one behavior on consecutive days for social, open field, hole board, light/dark, and EPM tasks. We then allowed 2 days between EPM and PPI, and between PPI and fear conditioning/recall (Figure 1).

### 4.2 Social preference/novelty

Mice were tested in a custom-made plastic three-chamber arena (24” × 16” × 9”) to study their propensity for social interaction and social novelty. Each chamber was (8” × 16” × 9”) with doors to allow free access between chambers. The center chamber remained empty throughout the test, but each of the sides contained an overturned pencil cup in one of the corners. The experiments were conducted in three phases, each being recorded by video for animal tracking and analysis. First, the mouse was allowed to investigate the whole apparatus in a 10 min habituation phase. Next, during the social preference phase, an age-matched, conspecific, non-familiar mouse was placed inside one of the overturned pencil cups in a side chamber while the other pencil cup remained empty. The test mouse was reintroduced to the apparatus and allowed to freely explore the three chambers for 10 min. Finally, the social novelty phase consisted of placing a second age-matched, conspecific, non-familiar mouse in the opposite pencil cup in the other side chamber and observing the test mouse for another 10 min. Each video was analyzed off-line using AnyMaze animal tracking software (Stoelting, Woods Dale, IL, USA). Social preference for the first mouse stage was measured as the total time the test mouse spent interacting with the mouse-in-a-cup compared to the empty cup as follows:

Equation 1 – social preference ratio


(1) $$S\,P\,R=\frac{\left(t_{m}-t_{e}\right)}{\left(t_{m}+t_{e}\right)}$$

where *SPR* is the social preference ratio, *t*_*m*_ is time spent interacting with the mouse-in-a-cup, and*t_*e*_* is time spent interacting with the empty cup. The preference ratio for social novelty was measured as the time spent in the chamber with the new mouse compared to the chamber with the mouse that was introduced in the previous phase as follows:

Equation 2 – social novelty ratio


(2) $$S\,N\,R=\frac{\left(t_{n}-t_{f}\right)}{\left(t_{n}+t_{f}\right)}$$

where *SNR* is the social novelty ratio, *t*_*n*_ is the time spent interacting with the novel mouse, and *t*_*f*_ is the time spent interacting with the familiar mouse.

### 4.3 Open field test

General locomotion and activity were tested in a clear plastic open field chamber (18” × 18” from AnyBox by Stoelting, Woods Dale, IL, USA). Mice were placed in the apparatus and allowed to freely explore for 10 min. All trials were recorded on video then analyzed off-line using AnyMaze animal tracking software.

### 4.4 Hole board test

To test restrictive or repetitive motion, a hole board (from AnyBox) was inserted into the open field chamber. The board covered the entire 18” × 18” box and consisted of 16 holes (1” in diameter) equally spaced in a 4 × 4 grid. A mouse was placed in the chamber and allowed to freely explore for 10 min. Infrared beam motion detectors were set up just below the level of the holes to detect investigative nose-pokes into a particular hole. All trials were recorded on video then analyzed off-line using AnyMaze animal tracking software.

### 4.5 Light/dark test

The 18” × 18” light/dark chamber (from AnyBox) is divided into two sections. One side (9” × 18”) has four opaque black walls and an opaque black lid which blocks the light from the room and stays quite dark while the adjoining side (9” × 18”) has three clear walls, sharing one of the black opaque walls, and is illuminated fully. A small opening in the joining wall allows for the mouse to freely move between the light portion and the dark portion of the box. Mice were placed in the light side of the box and allowed to freely explore for 10 min. All trials were recorded on video then analyzed off-line using AnyMaze animal tracking software.

### 4.6 Elevated plus maze

Another test for anxiety-like behavior is the EPM. A custom-built plus sign maze with two open arms (2” × 10”) and two walled arms (2” × 10” × 6.5”) stands 20” above the ground. Mice were placed on an open arm and allowed to freely explore the maze for 10 min. All trials were recorded on video then analyzed off-line using AnyMaze animal tracking software by Stoelting.

### 4.7 Acoustic startle response

The SR-LAB startle response system from San Diego Instruments (San Diego, CA, USA) consists of a control box with multiple IO ports for lights and cues, a 12” × 12” × 12” sound reducing chamber (for external noise) with a built in speaker, and tube-like enclosure for mice with an accelerometer attached to the bottom. We calibrated the analog output from the system to the speaker using a sound level meter (Extech Instruments, Waltham, MA, USA) to generate a curve of acoustic stimuli at appropriate noise levels. Mice were first habituated to the chamber during a 5-min session with background 65 dB white noise. Next, they were introduced to a series of 40 ms acoustic stimuli consisting of 8 pulses each of 70, 75, 80, 90, 100, and 110 dB white noise as well as 12 pulses each of 65 and 120 dB white noise interleaved in randomized order with randomized inter-pulse intervals (between 10 and 30 s). Startle responses measured by the accelerometer were averaged for each of the dB levels in order to build an input/output curve of acoustic sensitivity for each mouse. Four pulses of 120 dB noise were introduced at the beginning and end of the test to measure startle desensitization.

### 4.8 Pre-pulse inhibition

Pre-pulse inhibition tests were conducted with the SR-LAB startle response system (San Diego Instruments, San Diego, CA, USA). Mice were allowed to habituate to the chamber with 65 dB background noise for 5 min prior to testing. Mice were then introduced to a series of acoustic stimuli interleaved in randomized order with randomized inter-pulse intervals. Test pulses were 40 ms of 120 dB white noise that were preceded by pre-pulses of 20 ms long white noise at 70, 75, or 80 dB with either a 50 or 100 ms delay between the pre-pulse and the test pulse. Each pulse condition was introduced 6 times in random order, along with 12 test pulses with no pre-pulse condition and 12 background noise pulses of 65 dB, for a total of 60 acoustic stimuli. Four pulses of 120 dB noise were introduced at the beginning and the end of the test to measure startle desensitization. The accelerometer measured startle responses to each stimuli condition and was averaged for each mouse and the ratio of startle to the noise without a pre-pulse vs. those with a pre-pulse was used to find the level of PPI at each sound level according to the formula:

Equation 3 – pre-pulse inhibition


(3) $$P\,P\,I=1-\frac{r_{i}}{r_{m}}$$

where r_*i*_ is the average startle response to the inhibiting pre-pulse stimuli and r_*m*_ is the average startle response to the maximum startling stimuli (120 dB with no pre-pulse).

### 4.9 Fear conditioning

Differential auditory fear conditioning tests were conducted using a Med Associates (Fairfax, VT, USA) fear conditioning chamber (10” × 11.5” × 8.5”) inside a sound attenuating box (25” × 30” × 17”). During conditioning, two different acoustic stimuli were played to the mice and one was paired with a mild foot shock. The tone that was not paired with the shock (CS−) was a constant 30 s pure tone of 5,000 Hz at a level of 85 dB. The conditioned stimulus paired with the foot shock (CS+) was a series of 5 ms pure tone “pips” of 12,000 Hz at a level of 85 dB that occurred once a second for 30 s. This CS+ was co-terminated with a mild 1-s electric current of 0.8 mA (US) delivered through the grated floor of the chamber. Both the CS− and CS+/US were interleaved and delivered seven times with randomly variable inter-stimulus intervals. The behavior of the mice was recorded in the dark with an infrared camera (Basler, through Med Associates) and analyzed using Med Associates’ Video Freeze software.

### 4.10 Fear recall

Differential fear recall was conducted in the same Med Associates apparatus as the fear conditioning; however, to isolate the fear memory related to the acoustic stimulus alone and not the context of the box, we slightly modified the chamber. A plastic insert was laid on the floor of the chamber over the metal grating to change the tactile stimuli presented to the mouse, a plastic “tent” was placed above the mouse that spanned the whole of the chamber so that the shape of the box became a triangle, and all pieces were cleaned using 0.1% Alconox detergent (Alconox, White Plains, NY, USA) instead of 70% ethanol so that the olfactory signals would also be different. Mice were then re-presented with four each of the CS+ (without the US shock) and CS− acoustic stimuli interleaved at random inter-stimulus intervals and their freezing was recorded in the dark with the same infrared camera and Med Associates Video Freeze software. We measured the percentage of freezing that occurred during each of the 30-s epochs in which the tones were played.

### 4.11 Viral injections

Viruses were obtained from Vigene Biosciences (Rockville, MD, USA) and Addgene (Waterton, MA, USA). For local circuit mapping experiments, a soma-restricted version of Chrmine was combined with GCaMP6m using a P2A sequence so that infected cells expressed the opsin and Ca2+ indicator at the same time (AAV1-CAG-GCaMP6m-P2A-ChRmine-Kv2.1-HA). A total of 150 μl of this construct was injected into each hemisphere of the CeL (from Bregma: −1.34 AP, ±3.05 ML, 4.82 DV) and allowed to express for 3 weeks prior to the experiment. In a separate set of experiments, we injected ChR2 (AAV5-hSyn-hChR2(H134R)-EYFP) into the BLA (from Bregma: −1.75 AP, ±3.50 ML, 4.92 DV), waited 3 weeks for expression, and recorded photostimulated inputs onto CeL SOM+ cells. Surgeries were performed using a semi-robotic stereotaxic system, StereoDrive (Neurostar, Tubingen, Germany). Anesthesia was induced in an induction chamber via inhalation of 5% isoflurane (Isospire, Dechra, Northwich, UK) in O_2_ and maintained at 2% isoflurane in O_2_ using a SurgiVet Isotec Vaporizer (ICU Medical, San Clemente, CA, USA) calibrated by Sterling Biomedical (Boca Raton, FL, USA) at a flow rate of 0.8–1.0 L/min O_2_.

### 4.12 Electrophysiology

Mice were anesthetized in an induction chamber using 5% isoflurane in O_2_ and euthanasia was completed by cardiac perfusion of ice-cold cutting solution containing (in mM): 124 Choline Cl, 2.5 KCl, 26 NaHCO_3_, 1.25 NaH_2_PO_4_, 3.3 MgCl_2_, 10 Glucose, 0.5 CaCl_2_, with osmolarity adjusted to 295–305 mOsm (VAPRO Osmometer, ELITechGroup, Puteaux, France) and bubbled with 95% O_2_/5% CO_2_ (Airgas, Radnor, PA, USA). The brain was then removed, placed in ice-cold cutting solution and sliced at 350 μm on a Leica VT 1000 S Vibratome (Leica, Wetzlar, Germany). Slices were then placed in ACSF warmed to 37°C in a hot water bath and bubbled with 95% O_2_/5% CO_2_. The ACSF contains (in mM): 124 NaCl, 3 KCl, 26 NaHCO_3_, 1.25 NaH_2_PO_4_, 1 MgCl_2_, 20 Glucose, 5 Na-Ascorbate, 3 Na-Pyruvate, and 2 Thiourea and was adjusted to 295–305 mOsm. After 30 min incubation, slices were removed from the hot water bath in the same solution and allowed to come to room temperature.

Slices were then taken to an upright Zeiss Examiner Z1 microscope (Zeiss, Oberkochen, Germany) for whole-cell patch clamp experiments. We used a combination of the following Zeiss objectives (Zeiss, Oberkochen, Germany): 10x /0.3 NA W N Achroplan, 20x /1.0 NA W Plan Apochromat, and 40x /1.0 NA W Plan Apochromat. Glass micropipettes were fabricated using a Sutter P-97 micropipette puller (Sutter, Novato, CA, USA) with a resistance of 4–7 MOhms. We used Scientifica Microstar (Scientifica, Uckfield, UK) and Sutter MPC-200 (Sutter, Novato, CA, USA) micromanipulators on different rigs. All data were acquired at 20 kHz and low-pass filtered at 2 kHz using Clampex Software (pCLAMP 10), a Multiclamp 700B amplifier, and a Digidata 1440A digitizer (Molecular Devices, San Jose, CA, USA).

Cesium-based internal solutions were used, including Cs-Methanesulfonate (135 CsMeth, 6 NaCl, 10 HEPES, 0.6 EGTA, 4 MgATP, 0.3 Na_2_GTP, adjusted to 295 mOsm, and pH 7.25) and Cs-Gluconate (135 CsGlu, 8 NaCl, 10 HEPES, 0.2 EGTA, 10 CsCl, 2 MgATP, 0.2 Na_2_GTP, adjusted to 295 mOsm, and pH 7.25). Additional, various channel blockers were used as needed, including 20 μM Bicuculine, 10 μM NBQX, 50 μM AP5, and 1 μM TTX.

For the stimulation of afferent, channelrhodopsin-expressing terminals from the BLA onto CeL SOM+ neurons, we used a CooLED pE-100 illumination system (CoolLED Ltd., Andover, United Kingdom). The wavelength was set to 470 nm and the timing of activation was controlled through the digital outputs feature in Clampex.

### 4.13 Two-photon circuit mapping

For 2P experiments, the surgical procedures, solutions, microscope and electrophysiological equipment remained the same as above. We used one Ti:Sapph MaiTai laser (Spectra Physics-Newport, Irvine, CA, USA) tuned to 800 nm for imaging and one Ti:Sapph MaiTai DeepSee laser (Spectra Physics-Newport, Irvine, CA, USA) tuned to 1,020 nm for stimulation. The two light paths were controlled by two galvo-galvo mirror sets in the scanhead (Bruker, Billerica, MA, USA) and combined using a 970 shortpass dichroic (Chroma, Bellows Falls, VT, USA). Reflected fluorescent light was deflected toward the PMTs by a 700 nm longpass dichroic (Chroma, Bellows Falls, VT, USA) and separated using a dichroic filter cube with a 550 nm beamsplitter, 500–530 nm bandpass GFP filter and 570–620 nm TdTomato filter (Chroma, Bellows Falls, VT, USA). Gallium-Arsenic-Palladium photomultiplier tubes (GAsP PMTs) (Model H7421, Hamamatsu, Hamamatsu City, Japan) were used to amplify fluorescent signals. 2P mapping experiments were conducted under the 20*X* objective with a FOV of ∼405 × 405 μm.

To map local inhibitory circuits in the CeL, we used a combination of electrophysiology and 2P imaging. Post-synaptic cells were identified visually by the presence or absence of TdTomato. Such cells were patched under DIC in the whole-cell patch clamp configuration and held at 0 mV with NBQX and AP-5 to block excitatory neurotransmission and isolate inhibitory post-synaptic currents. We then switch to two-photon imaging to identify individual potential pre-synaptic partners by the expression of GCaMP6m and target them for photoactivation. Spiral scanning activation (8.5 μm diameter, 5 ms duration, 10 revolutions/spiral, 5 repetitions, and 1,020 nm stimulation laser) was used to stimulate putative pre-synaptic cells expressing ChRmine. The voltages applied to the galvo mirrors of the simulation laser corresponded to different pixel locations for the presynaptic targets and these were exported as a galvo points list (.gpl) file. Each cell was stimulated in succession with two frames of imaging between them such that an imaging sequence contained 3*n* + 25 frames (where *n* is the number of presynaptic targets, with 25 extra frames for baselines). Notably, 512 × 512 pixel images were obtained at a framerate of 1.3 Hz, however, we shuttered the PMTs for 50 ms around each spiral scan activation using a custom-made Arduino program. Four repetitions of each image sequence were collected for each FOV of each whole-cell patched post-synaptic cell. We controlled the 2P lasers and the imaging and activation parameters using Prairie View software (Version 5.5, Bruker, Bellerica, MA, USA).

### 4.14 Data analysis

Analysis of electrophysiological and imaging data was conducted using custom written MATLAB codes (available upon reasonable request). In brief, for general electrophysiological experiments, the recorded traces were imported, baselined, and averaged according to the number of repetitions. Peaks were identified and exported to excel for further examination and concatenation.

For circuit mapping experiments, the analysis consisted of four parts: processing imaging sequences, processing electrophysiological signals, identifying SOM+ cells, and bringing all the data together. Imaging sequences for each FOV of each post-synaptic cell consisted of, for an example of 100 presynaptic targets, 4 repetitions of 325 images, each 512 × 512 pixels. Image stacks were imported into FIJI (NIH, Bethesda, MD, USA) and separated into fluorescent channels. The green GCaMP stack was saved as a TIFF to be imported into MATLAB (Mathworks, Natick, MA, USA), while the red TdTomato stack was subjected to a median intensity projection and saved as a single image. In MATLAB, each repetition of the green GCaMP fluorescence for each FOV was averaged together and frames with the blank stimulation artifact were removed. We then used the GPL file to create a circular pixel mask 15 μm in diameter for each presynaptic target and applied it to the imaging stack to extract the GCaMP6 fluorescence for each presynaptic target cell independently. A pre-synaptic target was considered to have had a spike if the corresponding region of interest (ROI) showed an increase in fluorescence within five frames of activation that was greater than the baseline plus three times the standard deviation of the ROI, according to the formula:

Equation 4 – threshold for confirmation of presynaptic spiking via GCaMP6m fluorescence


(4) $$F_{n\,\ldots \,n+5}>F\,b\,a\,s\,e+3\,\left(\sigma \,\left(F_{n-10\,\ldots \,n-1}\right)\right)$$

where *Fbase* is the baselined fluorescence of a particular frame, *n* is the frame corresponding to the presynaptic target, and *σ(F_*n–10*…*n–1*_)* represents standard deviation of the 10 preceding baseline frames. Pre synaptic targets that did not surpass this threshold for Ca^2+^ activity were excluded from further analysis.

Electrophysiological signals were processed similar to other experiments. However, once the traces and peaks had been identified, we viewed each repetition separately to see responses on a trial-by-trial basis. A pre-synaptic cell was considered to be connected to the post-synaptic cell if in at least two of the four repetitions, a clear and obvious post-synaptic inhibitory current (IPSC) was observed.

Next, we opened the red TdTomato image for each FOV in FIJI and, using a custom ImageJ macro, overlaid the positions of each targeted cell’s ROI (generated in the same MATLAB code that translated the GPL voltages to pixel locations) and numbered them according to their order of stimulation. Then we manually identified if the cell within each ROI was a SOM+ cell based on the expression or lack of TdTomato.

We then brought all of this data together in excel to compute various parameters such as connection probability between and within cell types as well as the relationship between post synaptic current and the distance from the pre synaptic cell body. Connection probability was computed as:

Equation 5 – connection probability


(5) $$p=\frac{n_{c}}{n_{a}}$$

where *n*_*c*_ is the total number of presynaptic targets that showed obvious inhibitory post synaptic currents in addition to GCaMP fluorescence and *n*_*a*_ is the total number of presynaptic targets that had GCaMP fluorescence that passed the threshold for time-locked activity.

To determine the spatial distribution of pre synaptic cells and their relative contribution to the post synaptic currents recorded, we computed various current and distance relationships. We concatenated all the IPSC peaks, with the soma to soma distance for their corresponding pre synaptic ROI, for each stimulation pair (SOM+ to SOM+, SOM− to SOM+, etc.) and data together based on distance from the recorded cell. We computed a curve for the average current within each 20 μm bin as follows:

Equation 6 – distribution of average current by soma to soma distance


(6) $$I_{c}=\frac{\sum_{i=d-20}^{d}x_{i}}{n_{c}}$$

where *I*_*c*_ is the average current per cell for each bin, *x*_*i*_ is an individual IPSC peak, *d* is the top bound of the 20 μm bin for soma to soma distance, and *n*_*c*_ is the total number of post synaptic cells recorded in that configuration (SOM+ to SOM+, SOM− to SOM+, etc.). Graphs and figures were created and statistical analysis conducted using GraphPad Prism (Graphpad Software, Inc., San Diego, CA, USA).

## Data Availability

The raw data supporting the conclusions of this article will be made available by the authors, without undue reservation.
